# SOLID PSEUDOPAPILLARY NEOPLASM OF THE PANCREAS

**DOI:** 10.1590/0102-6720201600020007

**Published:** 2016

**Authors:** Jorge Roberto Marcante CARLOTTO, Franz Robert Apodaca TORREZ, Adriano Miziara GONZALEZ, Marcelo Moura LINHARES, Tarcisio TRIVIÑO, Benedito HERANI-FILHO, Alberto GOLDENBERG, Gaspar de Jesus LOPES-FILHO, Edson José LOBO

**Affiliations:** 1 Surgical Gastroenterology Division, Department of Surgery, School of Medicine, Federal University of São Paulo (EPM/UNIFESP); 2 Study Group of Pancreatic Diseases, São Paulo, SP, Brazil.

**Keywords:** Pancreatic neoplasms, Pancreatectomy, Pancreas

## Abstract

**Background:**

The solid pseudopapillary neoplasm is a rare tumor of the pancreas. However, it´s etiology still maintain discussions.

**Aim:**

To analyze it´s clinical data, diagnosis and treatment.

**Methods:**

A retrospective study of medical records of all patients treated from January 1997 until July 2015.

**Results:**

Were identified 17 cases. Most patients were women (94.11%) and the average age was 32.88 years. The main complaint was abdominal mass (47.05%). The most frequent location was in the body/tail of the pancreas (72.22%) and the most frequently performed surgery was distal pancreatectomy with splenectomy (64.70%). No patient had metastases at diagnosis. Conservative surgery for pancreatic parenchyma was performed in only three cases. The rate of complications in the postoperative period was 35.29% and the main complication was pancreatic fistula (29.41%). No patient underwent adjuvant treatment.

**Conclusions:**

The treatment is surgical and the most common clinical presentation is abdominal mass. Distal pancreatectomy with splenectomy was the most frequently performed surgery for its treatment.

## RESUMO

**Racional:**

A neoplasia sólida pseudopapilar é tumor raro de pâncreas de tratamento cirúrgico. No entanto, sua causa ainda gera discussões.

**Objetivo:**

Analisar os dados clínicos, do diagnóstico e do tratamento da dessa neoplasia.

**Métodos:**

Estudo retrospectivo com dados médicos de pacientes tratados entre janeiro de 1997 a julho de 2015.

**Resultados:**

Foram identificados 17 casos. A maioria era de mulheres (94,11%) e a média de idade foi de 32,88 anos. A principal queixa era massa abdominal (47,05%). A localização mais frequente era no corpo/cauda do pâncreas (72,22%) e a operação mais realizada foi a pancreatectomia corpocaudal com esplenectomia (64,70%). Nenhum caso apresentou metástase no momento do diagnóstico. Operação conservadora de parênquima pancreático foi realizada em apenas três casos. A taxa de complicações no pós-operatório foi de 35,29% e a principal complicação foi fístula pancreática (29,41%). Nenhum paciente realizou adjuvância no seguimento.

**Conclusões:**

A mais comum apresentação clínica da neoplasia sólida pseudopapilar é de massa abdominal. Ela é de tratamento cirúrgico e pancreatectomia corpocaudal com esplenectomia é o procedimento mais realizado para seu tratamento.

## INTRODUCTION

The solid pseudopapillary neoplasm (SPN) was first described by Frantz in 1959 and included in the World Health Organization classification in 1996^1^. The malignant potential is low and its pathogenesis is uncertain, what still motivates discussions. Corresponds to 0.17 to 3% of all malignant pancreatic cancers and affects mainly young women between the third and fourth decades of life^6^ ^,^ ^19^ ^,^ ^20^. The treatment is surgical resection and is associated with good results, favorable prognosis and the operative mortality is estimated at 2%^16^. Currently there are approximately 800 cases reported in the literature, the most part of the cases were limited to reports and case series due to its low incidence^11^ ^,^ ^18^ ^,^ ^24^.

The aim of this study is to analyze the clinical data, diagnosis and treatment of SPN.

## METHODS

A retrospective study of medical data obtained from medical records and database of all SPN treated in Surgical Gastroenterology Division of the School of Medicine, Federal University of São Paulo, São Paulo, SP, Brazil from January 1997 to July 2015. Data related to the preoperative, intraoperative and postoperative were collected. Information included age, gender, symptoms, imaging method for the diagnosis, tumor location in the pancreatic parenchyma and as its size. In addition, were analyzed the type of operations, postoperative complications, discharge and follow-up.

 The symptoms were defined as the main complaint of the patient. For the diagnosis of pancreatic fistula were adopted the recommendations of the International Study Group on Pancreatic Fistula Definition, characterized by measuring drainage fluid amylase from the third postoperative day with values greater than three times the normal limit^5^.

Data were collected and organized in Excel (Microsoft, USA). Statistical analysis of data was performed by SPSS 20.0 (IBM, USA). Quantitative variables were presented as mean±standard deviation and qualitative variables as frequency and percentage.

## RESULTS

Were identified in this period 17 patients treated with SPN. Most were women (94.11%) with a mean age of 32.88±11.86 years. The most frequent symptom was abdominal mass (47.05%). All patients underwent abdominal ultrasound, but 70.58% need to perform CT scan or MRI to confirm the diagnosis and in 72.22% the lesion was located in the body or pancreatic tail. No patient had metastases at diagnosis. The largest average diameter of the lesion was 6.52±3.01 cm. The injury-related data are displayed individually in Table 1.

**TABLE 1:** Data related to the diagnosis of SPN

Case	Age (years)	Gender	Symptoms	CT	RNM	Tumor location	Larger tumor size (cm)
1	51	F	Mass	Yes	Yes	Tail	6,5
2	22	F	Pain	Yes	No	Head	8
3	21	F	Mass	Yes	No	Body	6
4	22	F	Pain	Yes	Yes	Neck	5,4
5	50	F	Pain	Yes	No	Body	3,3
6	43	F	Pain	Yes	No	Body	6,2
7	32	F	Mass	Yes	No	Tail	5,7
8	24	F	Mass	Yes	Yes	Tail	11,5
9	35	F	Jaundice	Yes	Yes	Head	8,3
10	44	F	Mass	Yes	Yes	Tail	4,3
11	16	F	Mass	Yes	Yes	Body	13,5
12	32	F	Mass	Yes	Yes	Body	1,5
13	38	F	Mass	Yes	Yes	Body	7,3
14	49	M	Asymptomatic	Yes	Yes	Tail	8
15	39	F	Pain	Yes	Yes	Tail	5,3
16	24	F	Pain	Yes	Yes	Neck	2,4
17	17	F	Pain	Yes	Yes	Head/Body	4,8/7,8

F= Female. M= Male; CT= computed tomography; MRI= magnetic resonance imaging.

The diagnosis of SPN preoperatively was done in 82.35%, and all patients underwent surgical treatment. The distal pancreatectomy with splenectomy was the most performed operation (64.70%) and two of them were by laparoscopy. The mean operative time was 327±119 min. No patient had lymph node involvement. The most common postoperative complication was pancreatic fistula (29.41%), with four cases of type A and one case of type C. The hospital discharge occurred on average in 8.35±4,4 days postoperatively. The mean follow-up of 27.41± 23.74 months. There was one case of recurrence in patients underwent enucleation (5.88%) and one died (5.88%) on the third day after surgery due to thrombosis of the vascular graft performed by neoplastic involvement. Adjuvant therapy was not performed in patients (Table 2).

**TABLE 2:** Data related to the treatment of SPN

Case	Surgery	Duration (minutes)	Discharge (postoperative day)	Complications	Follow-up (months)
1	LPR - DP + S	250	5	Grade C fistula	78
2	PTD	325	21	Wound infection	83
3	DP + S	640	Death	Death	-
4	ENU	210	7	No	27
5	ENU	230	12	Grade A fistula	39
6	DP + S	320	8	No	31
7	DP + S	190	7	No	42
8	DP + S	270	11	No	39
9	PTD	490	8	No	26
10	DP + S	200	6	No	20
11	DP + S	235	7	No	15
12	LPR - DP + S	270	5	No	18
13	DP + S	450	7	No	21
14	DP + S	340	12	Grade A fistula	10
15	DP + S	370	10	Grade A fistula	12
16	Hybrid CP	380	10	Grade A fistula	3
17	STP + S	395	9	No	2

LPR=laparoscopic pancreatic resection; DP=distal pancreatectomy; S=splenectomy; PTD= pancreatoduodenectomy; ENU=enucleation. CP=central pancreatectomy; STP= subtotal pancreatectomy.

## DISCUSSION

Frantz described, in 1959, three patients with pancreatic tumor who had distinct characteristics of previously described pancreatic cancer cases, especially the presence of encapsulated lesion with cystic and solid areas. The solid component was structurally pseudopapillary which subsequently led to a number of denominations such as SPN, solid and cystic neoplasms, Frantz's and Hamoudi's tumor, among others, there is no consensus on the best denomination^8^. Since then, about 800 cases have been reported in the literature, their estimated impact comprises 0.3-2.7% of all pancreatic cancers. Unlike other tumors, it seems to remain stable in the last decades^11^ ^,^ ^18^ ^,^ ^24^. From the 80s, there was an increase in the number of cases of SPN identified and published and this fact probably linked to the progress of imaging methods and especially the histopathological and immunohistochemical study, which generated growing interest in the topic.

The largest case series published is Chinese, described by Cai et al., which presented 116 patients^6^. The greatest number of Brazilian cases due to Machado et al., had 34 patients^17^. Similarly to the above mentioned series, this shows the profile of these patients in university hospitals that for being reference, does not allow the analysis of real incidence of neoplasm. Table 3 lists the main case series published on the SPN in the last 22 years.

**TABLE 3:** Main case series in the world on SPN

	Salvia et al. (2007)	Machado et al. (2008)	Yang et al. (2009)	Cai et al. (2014)	Carlotto et al. (2016)
Number of cases	31	34	26	116	17
F	27 (87%)	27 (79%)	22 (85%)	100 (86%)	16 (94%)
M	4 (13%)	7 (21%)	4 (15%)	16 (14%)	1 (6%)
Mean age (years)	34	23	30	35	33
Main symptom	Pain	Pain	Pain	Pain	Mass
Incidental diagnosis	17(55%)	7 (21%)	11 (42%)	32 (27%)	1 (6%)
Location	BT (68%)	BT (61%)	HN (54%)	HN (53%)	BT (65%)
Mtx in diagnosis	0	0	0	5 (4%)	0
More frequent operation	DP + S	DP + S	PTD	DP	DP + S
Lymph nodes +	0	0	0	0	0
Pancreatic fistula	-	19 (56%)	3 (12%)	13 (11%)	5 (29%)
Adjuvant therapy	0	1 (3%)	0	0	0
Recurrence	0	0	1 (4%)	2 (2%)	1 (6%)
Death	0	0	1 (4%)	0	1 (6%)

F=female; M=male; BT=body/tail; HN=head/neck; Mtx=metastasis; DP=distal pancreatectomy; S=splenectomy; PTD= pancreatoduodenectomy.

The SPN characterize to affect young patients. Diagnosis usually occurs in the third decade of life^2^ ^,^ ^20^. In the main published case series, the average age at diagnosis ranged from 23-35^6^ ^,^ ^17^ ^,^ ^21^ ^,^ ^25^ and this series was 32.8 years, similar to the existing literature. Most cases occurred in women and there was only one case in men. The literature confirms this fact and the female-male ratio varies in proportion from 1.7-10:1^12^. This seems to be related to sex hormones such as estrogen and progesterone, but it is still unknown the true role of them with regard to the growth of the neoplasm or its histogenesis. Estrogen receptors are rarely present, whereas progesterone's are identified in most cases^17^ ^,^ ^22^. Tien et al. showed that there is no difference between genders in relation to the immunohistochemical profile and hormone receptor^22^. When men are affected by SPN, there is a tendency to the cancer begin in older age and more aggressive lesion^6^ ^,^ ^17^. Machado et al. and Cai et al. demonstrated through 7:16 cases, respectively, that this male gender was associated with a higher mean age at diagnosis, as well as larger tumor diameter^6^ ^,^ ^17^.

Treatment of SPN is surgical. It is a slow tumor progression and with good prognosis, but when they reach large proportions, may be associated with increased morbidity. Despite slow growth, some criteria are risk factors for poor prognosis. The criteria described in the literature are lesions larger than 5 cm, male, tumor necrosis, cellular atypia and vascular invasion, perineural and adjacent structures^9^ ^,^ ^10^ ^,^ ^15^. All patients in this study underwent surgery with complete resection of the lesion, although the largest average tumor diameter was 6.5 cm. Enucleation with preservation of pancreatic parenchyma was performed only in two patients (11.7%), and this circumstance is attributed mainly to the location of the lesion. This fact is also shown in the main case series, in which the enucleation of the lesion does not exceed 16% of the cases^6^ ^,^ ^17^ ^,^ ^21^ ^,^ ^25^. Even if SPN presents large, usually resection is possible and curative^23^. No patient in this series presented extrapancreatic invasion or distant metastasis. These findings are rare and not contraindicate resection, which must be completed and, if possible, monoblock^9^. Due to the slow nature of the neoplasm, vascular resection and when committed adjacent structures are shown and are indispensable for obtaining negative margins^6^ ^,^ ^8^. The lymphadenectomy is not necessary since the lymphatic spread is not part of the characteristics of this type of neoplasia^25^. In this series one of the patients required resection of the portal vein with vascular reconstruction because of extrapancreatic invasion of cancer. This fact added morbidity to the procedure, resulting in death in the postoperative third thrombosis of the vascular graft.

The laparoscopic surgery is also an option to the SPN and depends on the lesion location in the pancreatic parenchyma, tumor size and experience of the surgical team. The safety is the same as the open surgery, in addition to offering better surgical field, less pain after surgery and faster recovery of the patient^7^ ^,^ ^13^ ^,^ ^14^. Cavallini et al. demonstrated the efficacy of laparoscopy in 10 cases. There was no recurrence of the disease during follow-up and no deaths postoperatively^7^. In this series, there were two laparoscopic distal pancreatectomy with splenectomy and a hybrid central pancreatectomy in with small lesions. No local recurrence was identified in the follow-up of these patients treated by laparoscopy.

Pancreatic fistula is the most common complication after pancreatic resection, regardless of cause^3^ ^,^ ^4^ ^,^ ^5^ ^,^ ^26^. The most SPN lesions occur distally in the pancreas and in this location, the fistula has a better prognosis. The rate varies from 11-56% according to the literature^6^ ^,^ ^17^ ^,^ ^21^ ^,^ ^25^. This complication was present in 29.41% of cases and the most had no clinical impact on patient and did not require any intervention.

After surgical resection, about 95% of the patients were free of disease^1^ ^,^ ^15^ ^,^ ^19^ ^,^ ^21^. Only one patient had recurrence in the late postoperative follow-up, 17 months after enucleation of a pancreatic neck lesion. This recurrence is usually associated with patients with poor prognostic factors or incomplete resection of the disease. In addition to local recurrence, relapse can manifest through metastasis, and the liver as the primary site^20^ ^,^ ^23^. Treatment option in recurrence is adjuvant therapy, although there is no definitive evidence to suggest this treatment in SPN. Single cases with metastasis have been successfully treated by radiotherapy and chemotherapy with cisplatin, 5-fluorouracil and gemcitabine^11^ ^,^ ^17^.

## CONCLUSIONS

The main clinical sign of SPN was abdominal mass, which shows the late diagnosis and the large size of the lesion, making it difficult to perform operations that preserves pancreatic parenchyma. Treatment is surgical, but despite the favorable prognosis of the cancer, surgical treatment not always occurs without complications.
